# Prevalence and intensity of pain during diagnostic hysteroscopy in women attending an infertility clinic: analysis of 489 cases

**DOI:** 10.31744/einstein_journal/2020AO4916

**Published:** 2019-12-06

**Authors:** Andréa Pegoraro, Marcelo Ettruri Santos, Jean Tetsuo Takamori, Waldemar de Almeida Pereira de Carvalho, Renato de Oliveira, Caio Parente Barbosa, Ângela van Nimwegen

**Affiliations:** 1 Faculdade de Medicina do ABC, Santo André, SP, Brazil.

**Keywords:** Pain perception, Visual Analog Scale, Hysteroscopy, Anxiety, Infertility

## Abstract

**Objective:**

To investigate the prevalence and intensity of pain perception during diagnostic hysteroscopy in women and potential related factors.

**Methods:**

A total of 489 women were investigated at an infertility clinic. Fluid diagnostic hysteroscopy was performed without analgesia or anesthesia by gynecologists with different levels of experience in operative hysteroscopy, using a 2.9mm rigid scope. The Visual Analog Scale was used to score pain intensity after vaginal speculum insertion and after hysteroscopy. Data collected included age, ethnicity, body mass index, history of infertility and endometrial surgery (curettage and/or hysteroscopy), smoking habits, and hysteroscopy diagnosis. Only the state of anxiety was assessed by the State-Trait Anxiety Inventory given to each patient before the procedure.

**Results:**

Hysteroscopy median (25^th^ to 75^th^) Visual Analog Scale scored 3.3 (3 to 5), and 41.7% of the women referred Visual Analog Scale score ≥4. Median (25^th^ to 75^th^) State-Trait Anxiety Inventory score was 42 (38 to 45), and 58.3% of the women referred State-Trait Anxiety Inventory score >40. Hysteroscopy Visual Analog Scale score was significantly correlated to surgeon experience and to vaginal speculum insertion but not to State-Trait Anxiety Inventory score, ethnicity or abnormal hysteroscopic findings.

**Conclusion:**

Diagnostic hysteroscopy was mostly perceived as a mild discomfort procedure by most women. Nevertheless, in a considerable number of cases, women perceived hysteroscopy as painful. Pain perception was linked to individual pain threshold and surgeon experience, but not to pre-procedural anxiety state levels, ethnicity or abnormal hysteroscopic findings.

## INTRODUCTION

Hysteroscopy represents the gold standard for the evaluation of the uterine cavity and adequate endometrial sampling due to its minimal invasiveness and high diagnostic success rate.^1 , 2^ However, hysteroscopy remains painful, and approximately 30% of women referring considerable pain.^3^ Potential factors linked to pain perception during this procedure include scope diameter,^3^ medical experience, anxiety, and reproductive status.^4^

The use of mini-hysteroscopes (outer sheet diameter from 3 to 3.7mm) reduced significantly pain perception levels when compared to conventional 5mm devices. This technique has a less traumatic passage through the cervical canal and the internal part, leading to a less painful and better-tolerated examination. Nevertheless, thinner scopes failed to turn diagnostic hysteroscopy a painless procedure as some women still endure significant distress.^3^

The beneficial effect of medical experience on pain perception using conventional 5mm devices for diagnostic hysteroscopy is undeniable. However, some studies have shown that medical experience may lose its importance in reducing pain perception when the examination is performed with a mini-hysteroscope.^2 , 5^

Anxiety can enhance painful sensations at all levels of the nervous system, from the peripheral receptors to the cortical level.^6^ Anxiety before office hysteroscopy has been reported to have comparable levels to that by women undergoing gynecological surgery under general anesthesia. Nonetheless, the effect of anxiety on pain perception during diagnostic hysteroscopy has not been well defined yet.^7^

Pain is one of the limiting factors for the widespread use of hysteroscopy. To improve the quality of pain management and to evaluate new pain management techniques, pain must be measured, the results analyzed, and changes assessed for clinical significance. For the evaluation of pain intensity, a method commonly used is the Visual Analog Scale (VAS). This tool is easy to be used, the results are reproducible, and it can be applied in a variety of practical settings.^8^

The results could help patients who suffer with local anesthesia and/or impatience. This way, new measures to improve patient’s satisfaction could be implemented.

## OBJECTIVE

To investigate the prevalence and the intensity of pain perception during diagnostic hysteroscopy in women in a human reproduction service and to identify potential factors linked to it.

## METHODS

The study was approved by the local Research Ethics Committee in December 2013, under protocol number 489.536 and CAAE: 23033513.3.0000.0082. The patients had given their Informed Consent for participation in this study.

This observational study was performed with patients cared for at the Human Reproduction Service, in the city of Santo André (SP), Brazil, from February 2013 to September 2014. Inclusion criteria were women undergoing fertility investigation who chose to undergo diagnostic hysteroscopy at the clinic where the study was conducted. These women were randomly recruited in the waiting room. Only those who signed an Informed Consent Form participated.

Exclusion criteria were severe visual impairment, acute pelvic infection, inability to read and understand Portuguese language, and women who did not finish the procedure. These women were excluded once this research focused on the pain perception of patients who went through all phases of the procedure till the end.

In the waiting room, after signing the informed consent form, the participants were requested to complete a Portuguese version of the State-Trait Anxiety Inventory, (STAI-S).^9^ Only the state of anxiety was assessed. The STAI-S for adults consists of 20 self-reported items that measure anxiety state. An emotional state exists at a given moment in time and at a particular level of intensity. Anxiety state is characterized by subjective feelings of tension, apprehension, nervousness, and worry, and by activation or arousal of the autonomic nervous system. The participant recorded which one of four descriptors best indicated her degree of emotion: (1) not at all, (2) somewhat, (3) moderately so, and (4) very much so. Scores ranged from 20 to 80, and the higher the score the higher the anxiety level.^9^

Diagnostic hysteroscopy was performed without analgesia or anesthesia, by surgeons with different experience in operative hysteroscopy. Surgeons were categorized into 2 groups: experienced (1 qualified hysteroscopist with more than 500 operative procedures), and inexperienced (10 gynecologists who had performed less than 50 diagnostic hysteroscopies, supervised by 1 experienced hysteroscopist). Detailed clinical and demographic information were obtained from each participant during medical interview before starting the examination. Ethnicity was defined by the participant’s self-declared skin color/race, in compliance with the standard approach used to obtain official Brazilian statistics. The participant was positioned in the gynecological position. A small lubricated speculum was placed, and the vagina was disinfected with chlorhexidine. The women were requested to score pain perception related to speculum insertion using VAS. No tenaculum was used. A rigid optic (2.9mm rod optic lens, 30° Hopkins II, Karl Storz, Tuttlingen, Germany) with a 3.5mm single-flow sheath was placed in the external canal, and advanced under visual control after speculum removal. Saline solution at room temperature was used as distension medium, with a continuous flow and preset intrauterine pressure of 75mmHg, controlled by an electronic pump (Karl Storz Endoskope^®^, Hysteromat, Germany).

After examination, women were requested to score the intensity of pain by using a 10cm VAS. Pain rating according to a zero to 10 VAS (zero indicated no pain; 1 to 3, mild pain; 4 to 7, moderate pain, 8 to 10, severe pain) is recommended by World Health Organization (WHO) and Vancouver Island Health Authority. In our analysis, we took into account VAS >3 as indicative of pain.

Endometrial biopsies were performed with a Pipelle^®^ after pain scoring, if necessary. All hysteroscopic findings were recorded in a standardized electronic form. A complete visualization of the cervical canal, uterine cavity and tubal ostia, and the absence of any anatomical alterations were required to categorize the examination as normal. It was considered abnormal when any major or minor abnormalities, regardless of their clinical significance, were detected.

Qualitative data are presented as absolute and relative frequency, whereas quantitative data are presented as median and range (25^th^ to 75^th^) due to its abnormal distribution (Shapiro-Wilk test; p<0.05). Spearman’s correlation and Pearson’s χ^2^ tests were used to verify the relation between the intensity of pain perception and other variables in the sample. Non-parametric tests were used to compare the variables among the groups. Two-tailed p-values <0.05 were considered significant. All statistical analyses were performed with the software Stata version 11.0.

## RESULTS

Data from 489 out of 503 recruited women were included in the study. Fourteen cases were excluded due to examination failure to achieve a diagnosis for the following reasons: incomplete examination due to intolerable pain (9 cases), uterine bleeding (2 cases), insufficient visualization of uterine cavity (2 cases), and large polyp in the isthmus (1 case). The nine women that reported intolerable pain were rescheduled for an office hysteroscopy under sedation. These patients were initially included in the statistics, but we decided to exclude them for three reasons: (1) no hysteroscopy diagnosis was obtained since all patients asked to stop the procedure before uterine cavity being reached; (2) their pain threshold (VAS) may be comparable to the women that reported severe pain (VAS 8 to 10), but their pain tolerance was different; and (3) no changes were observed in the statistics after their withdrawal.

Patient age ranged from 19 to 56 years. The majority of the women had primary infertility, self-reported as white skin, non-smoker, overweight BMI, no history of endometrial surgery (curettage and/or hysteroscopy), and diagnostic hysteroscopy was performed in the proliferative phase. Most hysteroscopies were conducted by experienced surgeons with normal diagnosis. Detailed characteristics of the study population are presented in table 1 .

**Table 1:** Characteristics of the study population (n=489)

Characteristic	Median	Range (25^th^ to 75^th^)	
Age	35	22-48	
Body mass index	25	17-40	
Menstrual cycle day	11.2	9-12	
VAS hysteroscopy	3.3	3-5	
VAS speculum	0	0-7	
STAI-S score	42	38-45	
Surgeon, n (%)			
Experienced	391 (80)		
Inexperienced	98 (20)		
Infertility			
Primary	329 (67.3)		
Secondary	160 (32.7)		
Smoking, yes	34 (6.9)		
Previous uterine curettage, yes	56 (11.2)		
Previous hysteroscopy, yes	119 (24)		
Hysteroscopy diagnosis			
Normal	284 (58.1)		
Abnormal	205 (41.9)		
Intrauterine synechiae	32 (15.7)		
Endometrial polyps	110 (53.7)		
Focal endometrial thickness	28 (13.7)		
Endocervical stenosis/synechiae/polyp	3 (1.5)		
Submucosal myomas	22 (10.6)		
Mullerian malformation	10 (4.8)		
Ethnicity (self-reported skin color/ethnical origin)			
White	295 (60.33)		
Brown	143 (29.24)		
Black	43 (8.8)		
Asian	8 (1.63)		

VAS: Visual Analog Scale; STAI-S: State-Trait Anxiety Inventory.

**Figure 1:**
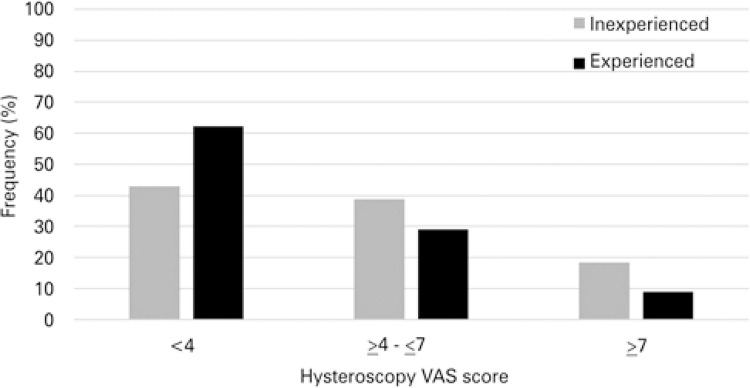
Prevalence of pain perception during diagnostic hysteroscopy according to categorized Visual Analog Scale score and surgeon experience VAS: Visual Analog Scale.

Median (25^th^ to 75^th^) VAS of the 489 women was 3.3 (3 to 5). Pain perception during hysteroscopy was not correlated to anxiety state, age or other clinical characteristics of the studied women. A correlation was detected with surgeon experience (Spearman’s correlation coefficient, r=0.2; p=0.001), with significantly higher VAS score in the inexperienced surgeon group (Mann-Whitney U Test, p=0.001). A positive correlation was also observed between VAS score of speculum insertion and VAS of hysteroscopy (Spearman’s correlation coefficient, r=0.3; p=0.001). Pain perception during hysteroscopy was then categorized in three groups according to VAS: (1) with <4,285 cases (58.3%); (2) ≥4 and ≤7, with 151 cases (30.9%); and (3) >7, with 53 cases (10.8%). The results showed that experienced surgeons had a higher proportion of women referring VAS <4, whereas inexperienced surgeons had a higher proportion of women referring VAS scores ≥4 and ≤7 or > 7 (Pearson χ^2^ test, p= 0.001; Figure 1 ). The results of the analysis between categorized VAS and other variables are presented in table 2 .

**Table 2:** Comparison of categorized Visual Analog Scale score with other variables

	VAS score			p value*^†^

	<4	≥4-≤7	>7	
	Median (CI95%)			
Age, years	34 (34-36)	35 (34.9-36)	34 (32-36)	0.175*
Body mass index	25 (24-26)	24 (23.9-25.0)	24 (23-26)	0.413*
STAI-S score	42 (41.39-42.61)	42 (41.09-42.91)	42 (40.55-43.45)	0.964*
Surgeon experience, n (%)				
Experienced	243 (62.2)	113 (28.9)	35 (9.0)	0.001^†^
Inexperienced	42 (42.9)	38 (38.8)	18 (18.4)	
Infertility, n (%)				
Primary	184 (55.6)	111 (33.5)	36 (10.9)	0.127^†^
Secundary	101 (63.9)	40 (25.3)	17 (10.8)	
Hysteroscopy diagnosis				
Normal	161 (56.7)	89 (31.3)	34 (12)	0.564^†^
Abnormal	124 (60.5)	62 (30.2)	19 (9.3)	
Previous hysteroscopy, n (%)				
No	220 (59.5)	115 (31)	35 (9.5)	0.218^†^
Yes	65 (54.6)	36 (30.3)	18 (15.1)	
Previous curettage, n (%)				
No	249 (57.4)	137 (31.6)	48 (11)	0.537^†^
Yes	36 (65.5)	14 (25.5)	5 (9.0)	
Ethnicity, n (%)				
White	176 (59.7)	93 (31.5)	26 (8.8)	0.52^†^
Brown	77 (53.8)	47 (32.9)	19 (13.5)	
Black	25 (58.1)	10 (24.4)	8 (19.5)	
Asian	7 (87.5)	1 (1.25)	0	

* Kruskal-Wallis test; ^†^ Pearson χ^2^ test.

VAS: Visual Analog Scale; STAI-S: State-Trait Anxiety Inventory; 95%CI: 95% confidence interval.

Median (25^th^ to 75^th^) STAI-S was 42 (38 to 45). A cutoff at 40 points was used to dichotomize STAI-S based on normal anxiety levels of Brazilian female population. The results showed that 58.3% of women referred STAI-S score >40. No association was found between anxiety state and the other variables in the studied population.

Endometrial polyp was the most prevalent abnormal hysteroscopic finding (110/284), followed by intrauterine synechiae (32/284), focal endometrial thickness (28/284), submucosal myoma (22/284), Mullerian malformation (10/284), and endocervical stenosis/synechiae/polyp (3/284). No correlation was observed between categorized VAS and these abnormal findings (Pearson χ^2^ test, p=0.482), even after grouping the findings in only two categories, as abnormal and normal (Pearson χ^2^ test, p=0.564).

## DISCUSSION

The present study primarily refers to the prevalence and intensity of pain experienced during diagnostic hysteroscopy in a selected population: women attending an infertility clinic. Indeed, the results showed that 41.7% of women referred VAS ≥4, which confirm hysteroscopy as a painful examination in a considerable number of cases. In previous studies with mixed population (different indications of hysteroscopy) a wide range of women referring VAS ≥4 was observed, varying from 21% to 88%, depending on several factors including reproductive status, distension medium and surgeon experience.^2 , 10 - 12^

Regarding the intensity of pain perception, the median VAS score across the 489 hysteroscopies was 3, suggesting the overall women perceived pain as a mild discomfort. A previous study with infertile population has reported slightly lower overall median VAS of 2.^11^ In studies with mixed populations, the mean VAS varied from 1.8 to 5.3.^2 , 13 - 15^ Considering only the 41,7% of women with VAS ≥4, the median VAS score was 6, revealing that these women underwent significant suffering. Paulo et al., in a recent systematic review and meta-analysis, stressed that pain is still a problem in hysteroscopy despite the paramount evolution observed in the last decades, and urged that investigation on its management should be continued.^3^ Pain, as defined by the International Association for the Study of Pain, is an unpleasant sensory and emotional experience that is associated with or described in terms of either potential or actual tissue damage. Its evaluation is now the so-called fifth vital sign, and its management is considered a fundamental human right.^16^

Potential factors associated with pain perception during hysteroscopy were investigated in the present study. The analysis identified two factors: pain during speculum placement and surgeon experience. Pain during speculum placement may be related to individual pain threshold. Pain threshold can be defined as the lowest intensity of painful stimulus at which the subject perceives pain. It is determined by a mosaic of neurobiological, cultural, and emotional factors,^17 , 18^ and its variability between individuals is prominent.^19^ For some women, speculum insertion is a disturbing and painful procedure of a gynecological examination, which can also be related to cultural factors and negative previous experience. Therefore, we speculate that pain during speculum placement may be linked to pain during hysteroscopy due to lower pain threshold in these women.

The impact of surgeon experience on pain perceived during hysteroscopy with small scopes is not well defined in the literature. We observed in the present study that experienced surgeons had a higher proportion of women referring VAS <4, whereas inexperienced surgeons had a higher proportion of women referring VAS ≥4. Other studies with infertile population also found that experienced surgeon is a protective factor for pain perception during diagnostic hysteroscopy.^11 , 20^ Conversely, some studies with mixed populations have suggested that mini scopes can counteract most of the difficulties determined by the uterine anatomy and by the operator, and consequently, makes it a less painful procedure.^2 , 5^

A secondary outcome of the present study is the women’s anxiety level before hysteroscopy. The median STAI-S was 42, which is higher than the score of the Brazilian female population (mean: 35.7).^9^ Moreover, the results showed that 58.3% of women referred STAI-S >40, which suggested a moderate level of anxiety before the examination in more than half of the cases. Other authors also confirmed moderate anxiety levels before diagnostic hysteroscopy. Carta et al., reported median STAI-S values of 41.50 in a sample of 94 women,^21^ whereas Kokanali et al., found mean STAI-S values of 44.8 (standard deviation: 10) in a sample of 148 women.^14^ This increased anxiety may be attributable to the expectation that invasive procedures will be performed in the outpatient setting and the fear of a serious underlying condition. Similar levels of anxiety have also been observed in other gynecological diagnostic procedures, such as mammography and colposcopy.^22 - 24^

The effect of anxiety state on pain perceived during hysteroscopy has also been addressed in the present study, and no correlation was observed between pain perception and anxiety state. Carta et al., also described similar results. The authors found a correlation between VAS score and waiting time, but not with STAI-S score.^21^ Conversely, Kokanali et al., demonstrated that preprocedural STAI-S score significantly affected VAS scores during and 60 minutes after hysteroscopy.^14^ Angioli et al., found that patients who listened to music during the procedure reported a lower VAS and a lower STAI-S. They suggested that anxiety state and pain perception are highly correlated. However, the correlation coefficient between VAS and STAI-S is not stated in their publication.^25^ To explain these conflicting results is difficult due to the heterogeneity of the studies. Instead, we would rather highlight their common finding: high prevalence of women presenting moderate levels of anxiety prior hysteroscopy. This is particularly important because anxiety can have repercussions on success of the procedure, as well as on overall patient experience and satisfaction.^7^ Implementation of non-pharmacological interventions, such as patient education, communication through traditional or multimedia approaches, music listening, interaction and support during the procedure are potential tools that can help reducing anxiety at hysteroscopy. Some evidence shows that nurses and nurse technicians play a relevant role in surgery-related anxiety reduction.^26^ Similarly, an improvement in pain thresholds and vaginal birth rates have been reported in obstetric research as a result of patient support by friends or doulas.^27^ It would be useful to attempt replicating those findings in the outpatient hysteroscopic setting.

In the present study, endometrial polyp was the most prevalence abnormal hysteroscopic finding followed by intrauterine synechiae, focal endometrial thickness, submucosal myoma and Mullerian malformation. The effect of these uterine abnormalities on perceived pain during hysteroscopy in women undergoing infertility investigation is unknown. It could be expected that women with these abnormalities would have more pain during hysteroscopy. However, our study found no association of polyps, intrauterine synechiae, myomas, endocervical stenosis or Mullerian malformation with pain. A study with mixed population also found similar results.^20^

The experience of pain is characterized by inter-individual and group variability with one likely contributing factor being ethnicity.^28^ Evidence exists for ethnic group differences in pain, with African Americans demonstrating greater severity of clinical and experimental pain.^29 , 30^ In the present study, however, no association was observed between patients’ ethnicity and perceived pain during hysteroscopy. Possible explanation for this result would be the complex mixed composition (Amerindian, European colonizers or immigrants, and African slaves) of the Brazilian population. A report from the EPIGEN-BRAZIL, using data from three Brazilian cohorts, each one from a different regions of the country (South, Southeast and Northeast), showed that these populations are genetically miscegenated at different levels, and that the patterns of association between self-reported skin color and genomic ancestry differ by site, probably because of the miscegenation level.^31^

The study has some limitations. Lack of information about imaging (ultrasound or magnetic resonance imaging) and patients’ history of chronic pelvic pain and dysmenorrhea limited the confirmation of these symptoms as predictive factor of pain during hysteroscopy. Actually, this needs to be further clarified since there is evidence suggesting that women affected by endometriosis and adenomyosis may show intense hyperalgesia during hysteroscopy, due to stimulation of sensitive nerve fibers at the level of endometrial functional layer.^32^ Another limitation was the lack of proper patients’ pain tolerance evaluation and satisfaction. It is worth noting that the term “pain tolerance” basically defines how much pain a person can actually take without breaking. It is influenced by people’s emotions, bodies, and lifestyles.^33^ We reported that 9 of 503 women experienced intolerable pain at hysteroscopy, and we have no plausible explanation for this intolerance.

## CONCLUSION

Diagnostic hysteroscopy was mostly perceived as a mild discomfort procedure. Nevertheless, in a considerable number of cases, women perceived hysteroscopy as a painful examination. Pain perception was linked to individual pain threshold and surgeon experience, but not to preprocedural anxiety state levels, ethnicity or abnormal hysteroscopic findings.
