# Supplementary material for: Prevalence and intensity of pain during diagnostic hysteroscopy in women attending an infertility clinic: analysis of 489 cases
Source: Einstein (Sao Paulo). 2019 Dec 6;18:eAO4916. doi: 10.31744/einstein_journal/2020AO4916 (PMC6905163; doi:10.31744/einstein_journal/2020AO4916)
Supplement: Translation

# Translation: Prevalência e intensidade da dor na histeroscopia diagnóstica em mulheres atendidas em uma clínica de infertilidade: análise de 489 casos

**Authors:** Andréa Pegoraro, Marcelo Ettruri Santos, Jean Tetsuo Takamori, Waldemar de Almeida Pereira de Carvalho, Renato de Oliveira, Caio Parente Barbosa, Ângela van Nimwegen

**Affiliations:** 1 Faculdade de Medicina do ABC, Santo André, SP, Brasil.

**Keywords:** Percepção da dor, Escala Visual Analógica, Histeroscopia, Ansiedade, Infertilidade

## Abstract

**Objetivo:**

Investigar a prevalência e a intensidade da percepção da dor durante a histeroscopia diagnóstica, bem como os possíveis fatores relacionados.

**Métodos:**

Foram incluídas 489 mulheres submetidas à propedêutica de infertilidade. A histeroscopia diagnóstica foi realizada sem analgesia ou anestesia, por ginecologistas com níveis de experiência diferentes em histeroscopia, usando histeroscópio rígido de 2,9mm. A Escala Visual Analógica foi utilizada para avaliar a intensidade da dor após a inserção do espéculo vaginal e após a histeroscopia. Os dados coletados incluíram idade, etnia, índice de massa corporal, história de infertilidade e cirurgia endometrial (curetagem e/ou histeroscopia), tabagismo e histeroscopia diagnóstica. Avaliou-se apenas o estado de ansiedade pelo Inventário de Ansiedade Traço-Estado de cada paciente antes do procedimento.

**Resultados:**

A mediana (25^º^a 75^º^) de histeroscopia pela Escala Visual Analógica foi 3,3 (3 a 5), e 41,7% das mulheres obtiveram pontuação ≥4. A mediana (25^º^a 75^º^) do Inventário de Ansiedade Traço-Estado foi 42 (38 a 45), e 58,3% das mulheres referiram pontuação >40. A pontuação da Escala Visual Analógica da histeroscopia apresentou correlação estatisticamente significante com a experiência do cirurgião e a inserção do espéculo vaginal, mas não a pontuação do Inventário de Ansiedade Traço-Estado, etnia ou achados histeroscópicos anormais.

**Conclusão:**

A histeroscopia diagnóstica foi percebida pela maioria das mulheres como desconforto leve, mas um número considerável de pacientes classificou o procedimento como doloroso. A percepção da dor esteve ligada ao limiar individual e à experiência do cirurgião, mas não aos níveis de ansiedade pré-procedimento, à etnia e nem aos achados histeroscópicos anormais.

## INTRODUÇÃO

A histeroscopia representa o padrão-ouro para avaliação da cavidade uterina e coleta de amostras do endométrio, pois é minimamente invasiva e apresenta altas taxas de sucesso diagnóstico.^1 , 2^ No entanto, a histeroscopia permanece um procedimento doloroso, sendo que aproximadamente 30% das mulheres referem dor considerável.^3^ Possíveis fatores relativos à percepção da dor durante o procedimento incluem o diâmetro do histeroscópio,^3^ a experiência do médico, o nível de ansiedade, e o estágio reprodutivo.^4^

O uso de mini-histeroscópios (diâmetro externo de 3 a 3,7mm) reduziu consideravelmente os níveis de percepção de dor em comparação aos dispositivos convencionais de 5mm. Essa técnica tem passagem menos traumática pelo canal cervical e na parte interna, resultando em exame menos doloroso e mais bem tolerado. Ainda assim, mesmo os histeroscópios mais finos, não conseguiram eliminar a dor da histeroscopia diagnóstica, e algumas mulheres continuam sentindo bastante desconforto.^3^

O efeito positivo da experiência do médico sobre a percepção da dor durante o exame com dispositivos convencionais de 5mm para histeroscopia diagnóstica é inquestionável. No entanto, alguns estudos demonstraram que a experiência do médico pode perder sua relevância na redução da percepção de dor quando o exame é realizado com mini-histeroscópio.^2 , 5^

A ansiedade pode acentuar a sensação de dor em todos os níveis do sistema nervoso, dos receptores periféricos ao nível cortical.^6^ Há relatos de que a ansiedade que antecede a histeroscopia no consultório médico ocorre em níveis comparáveis aos das mulheres submetidas a cirurgias ginecológicas sob anestesia geral. Contudo, o efeito da ansiedade na percepção da dor durante a histeroscopia diagnóstica ainda não foi bem delineado.^7^

A dor é um dos fatores limitantes do uso disseminado da histeroscopia. Para melhorar a qualidade do controle da dor e avaliar novas técnicas para seu controle, a dor deve ser medida; os resultados, analisados; e possíveis mudanças quanto a seu significado clínico devem consideradas.

Para avaliação da intensidade da dor, um dos métodos mais usados é a Escala Visual Analógica (EVA). A EVA é fácil de usar, seus resultados são reprodutíveis, e ela pode ser aplicada em uma variedade de cenários práticos.^8^

Os resultados podem ajudam pacientes que sofrem com a anestesia local e/ou impaciência. Dessa forma, é possível implementar novas medidas para melhorar a satisfação dos pacientes.

## OBJETIVO

Investigar a prevalência e a intensidade da percepção da dor durante histeroscopia diagnóstica em mulheres atendidas em um serviço de reprodução humana, e identificar possíveis fatores relacionados.

## MÉTODOS

O estudo foi aprovado pelo Comitê de Ética em Pesquisa em dezembro de 2013, protocolo 489.536, CAAE: 23033513.3.0000.0082. As pacientes deram seu consentimento para participar deste estudo.

Este estudo observacional foi realizado com pacientes atendidas em um Serviço de Reprodução Humana, em Santo André (SP), de fevereiro de 2013 a setembro de 2014. Os critérios de inclusão foram mulheres submetidas à propedêutica de fertilidade, que optaram pela realização da histeroscopia diagnóstica na clínica em que o estudo foi conduzido. Essas mulheres foram aleatoriamente recrutadas na sala de espera. Só participaram as mulheres que assinaram o Termo de Consentimento Livre e Esclarecido.

Os critérios de exclusão foram deficiência visual grave, infecção pélvica aguda, incapacidade de ler e compreender o idioma português e não ter concluído o procedimento − essas mulheres foram excluídas porque o foco deste estudo era a percepção de dor das pacientes submetidas a todas as fases do procedimento, até a conclusão.

Na sala de espera, após assinar o Termo de Consentimento, as participantes tiveram que preencher uma versão em português do Inventário de Ansiedade Estado-Traço (IDATE).^9^ Avaliamos apenas o estado de ansiedade. O IDATE para adultos consiste de 20 itens autorreportados que medem o estado de ansiedade. Um estado emocional ocorre em determinado momento no tempo e em determinado nível de intensidade. O estado de ansiedade é caracterizado por sentimentos subjetivos de tensão, apreensão, nervosismo e preocupação, e pela ativação ou excitação do sistema nervoso autônomo. A participante registrava qual dos quatro descritores era mais indicativo de seu nível de emoção: (1) nada, (2) um pouco, (3) moderadamente e (4) muito. As pontuações variaram de 20 a 80, e quanto maior a pontuação, maior o nível de ansiedade.^9^

A histeroscopia diagnóstica foi realizada sem analgesia ou anestesia, por ginecologistas com diferentes níveis de experiência em histeroscopia. Os cirurgiões foram classificados em 2 grupos: com experiência (1 histeroscopista qualificado, com mais de 500 procedimentos realizados), e sem experiência (10 ginecologistas com menos de 50 histeroscopias diagnósticas realizadas, sob supervisão de 1 histeroscopista experiente). Informações clínicas e demográficas detalhadas foram solicitadas de cada participante durante a entrevista médica, antes do início do exame. A etnia foi definida pela cor de pele/raça autorreportada da paciente, de acordo com o padrão usado nas estatísticas oficiais brasileiras. A participante foi colocada em posição ginecológica. Um pequeno espéculo lubrificado foi introduzido, e a vagina foi desinfetada com clorexidina. Pediu-se que as mulheres pontuassem sua percepção de dor relativa à inserção do espéculo usando a EVA. Não foi usada pinça. Uma lente rígida (de 2,9mm, 30° Hopkins II, Karl Storz, Tuttlingen, Alemanha) com bainha única de 3,5mm foi colocada no canal externo e avançada sob controle visual, após a retirada do espéculo. Usou-se solução salina em temperatura ambiente como meio de distensão, com vazão contínua e pressão intrauterina predefinida de 75mmHg, controlada por bomba eletrônica (Karl Storz Endoskope^®^, Hysteromat, Alemanha).

Após o exame, pediu-se que as mulheres pontuassem a intensidade da dor, usando uma EVA de 10cm. A avaliação da dor segundo a EVA de zero a 10 (zero corresponde a nenhuma dor; 1 a 3 a dor leve; 4 a 7 a dor moderada; 8 a 10 a dor grave) é recomendada pela Organização Mundial da Saúde (OMS) e pela *Vancouver Island Health Authority* . Em nossa análise, consideramos pontuação >3 na EVA como indicativa de dor.

Foram realizadas biópsias endometriais com Pipelle^®^após a avaliação da dor, caso fossem necessárias. Todos os achados histeroscópicos foram registrados em formulário eletrônico padrão. Visualização completa do canal cervical, cavidade uterina e óstios das tubas, e ausência de alterações anatômicas foram requisitos para classificar o exame como normal. O exame foi considerado anormal em caso de alterações maiores ou menores detectadas, independentemente de seu significado clínico.

Os dados qualitativos são apresentados na forma de frequências absoluta e relativa, enquanto os dados quantitativos são apresentados como mediana e variação (25^o^a 75^o^) devido à sua distribuição anormal (teste de Shapiro-Wilk; p<0,05). Os testes de correlação de Spearman e do χ^2^ de Pearson foram usados para verificar a relação entre a intensidade de percepção da dor e outras variáveis na amostra. Testes não paramétricos foram usados para comparar as variáveis entre os grupos. Valores bicaudais de p<0,05 foram considerados significativos. Todas as análises estatísticas foram realizadas no programa State versão 11.0.

## RESULTADOS

O estudo incluiu dados de 489 das 503 mulheres recrutadas. Foram excluídos 14 casos devido ao fato de que o exame não foi capaz de chegar a um diagnóstico, por algum dos seguintes motivos: exame incompleto devido à dor intolerável (9 casos), sangramento uterino (2 casos), visualização insatisfatória da cavidade uterina (2 casos) e presença de pólipo grande no istmo (1 caso). As nove mulheres que reportaram dor intolerável foram reconvocadas para uma histeroscopia sob sedação no consultório médico. Essas pacientes inicialmente foram incluídas na estatística, mas depois decidimos excluí-las, por três motivos: (1) nenhum diagnóstico foi definido na histeroscopia, já que todas essas pacientes pediram que o procedimento fosse interrompido antes que a cavidade uterina fosse visualizada; (2) o limiar de dor (EVA) dessas pacientes talvez seja comparável ao daqueles que reportaram dor grave (EVA 8-10), mas a tolerância à dor foi diferente; e (3) não houve mudança nas estatísticas após a retirada dessas mulheres do estudo.

A idade das pacientes variou entre 19 a 56 anos. A maioria das mulheres tinha infertilidade primária, reportaram-se como brancas, não fumantes, com índice de massa corporal de sobrepeso, sem histórico de cirurgia do endométrio (curetagem e/ou histeroscopia), e a histeroscopia diagnóstica foi realizada na fase proliferativa. A maior parte das histeroscopias foi feita por cirurgiões experientes, e o diagnóstico foi normal. As características detalhadas da população do estudo são apresentadas na tabela 1 .

**Tabela 1:** Características da população do estudo (n=489)

	Mediana	Variação (25^o^a 75^o^)	
Idade	35	22-48	
Índice de massa corporal	25	17-40	
Dia do ciclo menstrual	11,2	9-12	
EVA durante a histeroscopia	3,3	3-5	
EVA durante a inserção do espéculo	0	0-7	
Escore IDATE	42	38-45	
Cirurgião, n (%)			
Experiente	391 (80)		
Sem experiência	98 (20)		
Infertilidade			
Primária	329 (67,3)		
Secundária	160 (32,7)		
Fumante, sim	34 (6,9)		
Curetagem uterina prévia, sim	56 (11,2)		
Histeroscopia prévia, sim	119 (24)		
Diagnóstico na histeroscopia			
Normal	284 (58,1)		
Anormal	205 (41,9)		
Sinéquia intrauterina	32 (15,7)		
Pólipos endometriais	110 (53,7)		
Espessamento endometrial focal	28 (13,7)		
Estenose/sinéquia/pólipo endocervical	3 (1,5)		
Miomas submucosos	22 (10,6)		
Malformação mulleriana	10 (4,8)		
Etnia (cor da pele/origem étnica auto-reportada)			
Branca	295 (60,33)		
Parda	143 (29,24)		
Negra	43 (8,8)		
Asiática	8 (1,63)		

EVA: Escala Visual Analógica; IDATE: Inventário de Ansiedade Traço-Estado.

A pontuação mediana (25^o^a 75^o^) da EVA das 489 mulheres foi de 3,3 (3 a 5). A percepção de dor durante a histeroscopia não teve correlação com estado de ansiedade, idade ou outras características clínicas das mulheres estudadas. Foi detectada correlação com a experiência do cirurgião (coeficiente de correlação de Spearman, r=0,2; p=0,001), com pontuação significativamente mais alta na EVA para o grupo em que o cirurgião não tinha experiência (teste U de Mann-Whitney, p=0,001). Uma correlação positiva também foi observada entre a pontuação EVA da inserção do espéculo e a pontuação EVA da histeroscopia (coeficiente de correlação de Spearman, r=0,3; p=0,001). A percepção da dor durante a histeroscopia foi, então, categorizada em três grupos de acordo com a EVA: (1) <4, com 285 casos (58,3%); (2) ≥4 e ≤7, com 151 casos (30,9%); e (3) >7, 53 casos (10,8%). Os resultados mostraram que cirurgiões experientes tiveram maior proporção de mulheres que relataram EVA <4, enquanto os cirurgiões sem experiência tiveram maior proporção de mulheres que referiram EVA ≥ 4 e ≤7 ou > 7 (teste de Pearson χ^2^ , p= 0,001; Figura 1 ). Os resultados da análise entre a classificação da pontuação EVA e outras variáveis são apresentados na tabela 2 .

**Figura 1:** Prevalência da percepção de dor durante histeroscopia diagnóstica, de acordo com a classificação da pontuação na Escala Visual Analógica e a experiência do cirurgião EVA: Escala Visual Analógica.

**Tabela 2:** Comparação entre a classificação da pontuação na Escala Visual Analógica e outras variáveis

	Pontuação na EVA			Valor de p^*†^

	<4	≥4-≤7	>7	
	Mediana (IC95%)			
Idade, anos	34 (34-36)	35 (34,9-36)	34 (32-36)	0,175*
Índice de massa corporal	25 (24-26)	24 (23,9-25,0)	24 (23-26)	0,413*
Pontuação IDATE	42 (41,39-42,61)	42 (41,09-42,91)	42 (40,55-43,45)	0,964*
Experiência do cirurgião, n (%)				
Experiente	243 (62,2)	113 (28,9)	35 (9,0)	0,001^†^
Sem experiência	42 (42,9)	38 (38,8)	18 (18,4)	
Infertilidade				
Primária	184 (55,6)	111 (33,5)	36 (10,9)	0,127^†^
Secundária	101 (63,9)	40 (25,3)	17 (10,8)	
Diagnóstico na histeroscopia, n (%)				
Normal	161 (56,7)	89 (31,3)	34 (12)	0,564^†^
Anormal	124 (60,5)	62 (30,2)	19 (9,3)	
Histeroscopia prévia, n (%)				
Não	220 (59,5)	115 (31)	35 (9,5)	0,218^†^
Sim	65 (54,6)	36 (30,3)	18 (15,1)	
Curetagem prévia, n (%)				
Não	249 (57,4)	137 (31,6)	48 (11)	0,537^†^
Sim	36 (65,5)	14 (25,5)	5 (9,0)	
Etnia, n (%)				
Branca	176 (59,7)	93 (31.5)	26 (8,8)	0,52^†^
Parda	77 (53,8)	47 (32.9)	19 (13,5)	
Negra	25 (58,1)	10 (24,4)	8 (19,5)	
Asiática	7 (87,5)	1 (1,25)	0	

* Teste de Kruskal-Wallis;^†^teste do χ^2^de Pearson.

EVA: Escala Visual Analógica; IC95%: intervalo de confiança de 95%; IDATE: Inventário de Ansiedade Traço-Estado.

A pontuação mediana (25^o^a 75^o^) no IDATE foi 42 (38 a 45). Um ponto de corte de 40 pontos foi usado para dicotomizar a pontuação no IDATE com base nos níveis normais de ansiedade da população feminina no Brasil. Os resultados mostraram que 58,3% das mulheres referiram IDATE >40. Não houve associação entre o estado de ansiedade e outras variáveis na população estudada.

Pólipo endometrial foi o achado anormal mais prevalente à histeroscopia (110/284), seguido por sinéquias intrauterinas (32/284), espessamento endometrial focal (28/284), mioma submucoso (22/284), malformação mulleriana (10/284) e estenose/sinéquia/pólipo endocervical (3/284). Não houve correlação entre a categoria de pontuação na EVA e esses achados anormais (teste do χ^2^ de Pearson, p=0,482), mesmo após o agrupamento dos achados em apenas duas categorias, anormais e normais (teste χ^2^ de Pearson, p=0,564).

## DISCUSSÃO

Este estudo trata principalmente da prevalência e da intensidade da dor vivenciada durante a histeroscopia diagnóstica em uma população selecionada: mulheres atendidas em uma clínica de infertilidade. Os resultados mostraram que 41,7% das mulheres referiram EVA ≥4, confirmando que a histeroscopia é um exame doloroso em um número considerável de casos. Em estudos anteriores com população mista (diferentes indicações para histeroscopia), observou-se grande variação na proporção de mulheres que reportaram EVA ≥4, entre 21% e 88%, dependendo de diversos fatores, como estágio reprodutivo, meio de distensão e experiência do cirurgião.^2 , 10 - 12^

Em relação à intensidade da percepção de dor, a pontuação da EVA mediana de 489 histeroscopias foi 3, indicando que, no geral, as mulheres perceberam a dor como leve desconforto. Um estudo anterior com a população infértil apresentou mediana de pontuação global na EVA de 2, ou seja, discretamente mais baixa.^11^ Em estudos com populações mistas, a pontuação média na EVA variou de 1,8 a 5,3.^2 , 13 - 15^ Considerando apenas os 41,7% de mulheres com EVA ≥4, a mediana da pontuação na EVA foi 6, o que revela que essas mulheres passaram por sofrimento considerável. Paulo et al., em revisão sistemática e metanálise recentes, destacaram que a dor ainda é um problema na histeroscopia, apesar de toda a evolução observada nas últimas décadas, e reforçaram que a investigação de como tratá-la deve prosseguir.^3^ A dor, conforme definida pela *International Association for the Study of Pain* , é uma experiência sensorial e emocional desagradável, associada ou descrita em termos de danos reais ou potenciais aos tecidos. A dor agora é conhecida como o quinto sinal vital, e seu tratamento é considerado um direito humano fundamental.^16^

Possíveis fatores associados à percepção de dor na histeroscopia foram investigados neste estudo. A análise identificou dois fatores: dor durante a inserção do espéculo e experiência do cirurgião. A dor durante a colocação do espéculo pode estar relacionada ao limiar individual de dor. O limiar de dor pode ser definido como a menor intensidade de estímulo doloroso com a qual o indivíduo sente dor. É determinado por um mosaico de fatores neurobiológicos, culturais e emocionais,^17 , 18^ e sua variabilidade interindividual é acentuada.^19^ Para algumas mulheres, a inserção do espéculo é uma parte incômoda e dolorosa de um exame ginecológico, que pode também estar relacionada a fatores culturais e experiências negativas no passado. Portanto, acreditamos que a dor durante a inserção do espéculo pode estar relacionada à dor na histeroscopia, devido a um limiar de dor mais baixo nessas mulheres.

O impacto da experiência do cirurgião na percepção de dor durante a histeroscopia com pequenos histeroscópios ainda não foi bem definida na literatura. Observamos, neste estudo, que os cirurgiões experientes tiveram maior proporção de mulheres com EVA <4, enquanto os inexperientes tiveram maior proporção de mulheres com EVA≥4. Outros estudos com a população infértil também verificaram que a experiência do cirurgião é um fator de proteção para a percepção de dor durante histeroscopia diagnóstica.^11 , 20^ Por outro lado, alguns estudos com populações mistas sugeriram que os mini-histeroscópios conseguem compensar a maioria das dificuldades determinadas pela anatomia uterina e pelo operador e, sendo assim, tornar o procedimento menos doloroso.^2 , 5^

Um desfecho secundário deste estudo foi o nível de ansiedade das mulheres antes da histeroscopia. A pontuação mediana no IDATE foi 42, mais alta que a pontuação da população feminina do Brasil (média de 35,7).^9^ Além disso, os resultados mostraram que 58,3% das mulheres tiveram IDATE >40, o que indica nível moderado de ansiedade antes do exame em mais de metade dos casos. Outros autores também confirmaram níveis moderados de ansiedade antes da histeroscopia diagnóstica. Carta et al. reportaram pontuação mediana no IDATE de 41,50 em amostra de 94 mulheres,^21^ enquanto Kokanali et al., encontraram pontuação média de 44,8 (desvio padrão de 10) em amostra de 148 mulheres.^14^ Essa maior ansiedade pode ser decorrente da expectativa de se submeter a um procedimento invasivo no contexto ambulatorial e do medo de descobrir uma doença séria. Níveis semelhantes de ansiedade também foram observados em outros procedimentos diagnósticos ginecológicos, como mamografia e colposcopia.^22 - 24^

O efeito do estado de ansiedade na dor sentida na histeroscopia também foi abordado neste estudo, e não houve correlação entre a percepção de dor e o estado de ansiedade. Carta et al., também chegaram a resultados semelhantes. Os autores encontraram correlação entre o tempo de espera e a EVA, mas não o IDATE.^21^ Por outro lado, Kokanali et al., demonstraram que o IDATE pré-procedimento teve efeito significativo sobre a EVA durante e 60 minutos após o procedimento.^14^ Angioli et al., constataram que pacientes que ouviram música durante o procedimento reportaram pontuações mais baixas em EVA e IDATE. Sugeriram que o estado de ansiedade e a percepção de dor são altamente correlacionados. No entanto, o coeficiente de correlação entre EVAS e IDATE não foi descrito na respectiva publicação.^25^ É difícil explicar esses resultados contraditórios devido à heterogeneidade dos estudos. Em vez disso, preferimos destacar um achado em comum: a alta prevalência de mulheres com níveis moderados de ansiedade antes da histeroscopia. Isso é particularmente importante porque a ansiedade pode ter repercussões no sucesso do procedimento, bem como na experiência e na satisfação globais da paciente.^7^ A implementação de intervenções não farmacológicas, como educação das pacientes, comunicação por abordagens tradicionais ou multimeios, ouvir música, interação e suporte durante o procedimento são possíveis ferramentas que ajudam a reduzir a ansiedade na histeroscopia. Algumas evidências mostram que os enfermeiros e os técnicos de enfermagem têm papel relevante na redução da ansiedade relacionada a uma cirurgia.^26^ Da mesma forma, estudos de obstetrícia reportaram melhora nos limiares de dor e taxas de parto vaginal quando as pacientes contavam com o apoio de amigos ou doulas.^27^ Seria interessante tentar replicar esses achados no contexto ambulatorial das histeroscopias.

Neste estudo, pólipo endometrial foi o achado histeroscópico mais prevalente, seguido por sinéquias intrauterinas, espessamento endometrial focal, mioma submucoso e malformação mülleriana. O efeito dessas alterações uterinas na percepção de dor durante a histeroscopia em mulheres submetidas à investigação de infertilidade ainda é desconhecido. Seria esperado que mulheres com essas alterações sentissem mais dor na histeroscopia. Porém, nosso estudo não mostrou associação entre os pólipos, sinéquias intrauterinas, miomas, estenose endocervical ou malformação mülleriana e dor. Um estudo em população mestiça encontrou resultados semelhantes.^20^

A experiência da dor é caracterizada pela variabilidade interindividual e de grupo, e um dos fatores potencialmente relevantes é a etnia.^28^ Há evidências de diferenças entre grupos étnicos relativas à dor, sendo que os afro-americanos demonstram dor clínica e experimental de maior gravidade.^29 , 30^ Neste estudo, entretanto, não houve associação entre a etnia da paciente e a dor sentida durante a histeroscopia. Uma possível explicação para este resultado seria a composição miscigenada e complexa (ameríndios, colonizadores ou imigrantes europeus e escravos africanos) da população brasileira. Um relatório da EPIGEN-BRASIL, usando dados de três coortes brasileiras, cada uma de uma região diferente do país (Sul, Sudeste e Nordeste) mostrou que essas populações são geneticamente miscigenadas em diferentes níveis, e que os padrões de associação entre a cor da pele autorreferida e a ancestralidade genômica são diferentes para cada centro, provavelmente por causa desse nível de miscigenação.^31^

O estudo tem algumas limitações. A falta de informações sobre imagens (ultrassom ou ressonância magnética) e sobre qualquer possível histórico de dor pélvica crônica e dismenorreia limitou a confirmação desses sintomas como preditores de dor na histeroscopia. Isso precisa ser esclarecido mais a fundo, já que existem evidências indicando que as mulheres com endometriose e adenomiose podem apresentar hiperalgesia intensa na histeroscopia, devido à estimulação de fibras nervosas sensíveis no nível da camada funcional do endométrio.^32^ Outra limitação foi a falta de avaliação apropriada da tolerância à dor e da satisfação das pacientes. Vale lembrar que o termo “tolerância à dor” basicamente define quanta dor uma pessoa consegue suportar sem sucumbir. Isso é influenciado pelas emoções, pelo corpo e pelo estilo de vida das pessoas.^33^ Reportamos aqui que 9 de 503 mulheres sentiram dor intolerável na histeroscopia, e não temos explicação plausível para essa intolerância.

## CONCLUSÃO

A histeroscopia diagnóstica foi predominantemente percebida como um procedimento que resulta em desconforto leve. Entretanto, em um número considerável de casos, as mulheres consideraram a histeroscopia um procedimento doloroso. A percepção de dor teve conexão com o limiar de dor individual e experiência do cirurgião, mas não com os níveis de ansiedade pré-procedimento, etnia ou achados anormais ao exame.
